# Supplementary material for: TUBB3 Reverses Resistance to Docetaxel and Cabazitaxel in Prostate Cancer
Source: Int J Mol Sci. 2019 Aug 13;20(16):3936. doi: 10.3390/ijms20163936 (PMC6719236; doi:10.3390/ijms20163936)
Supplement: Supplementary file 1 [file ijms-20-03936-s001.zip › ijms-567404 sp for proof/Supplementary figure 1.pptx]

## Slide 1
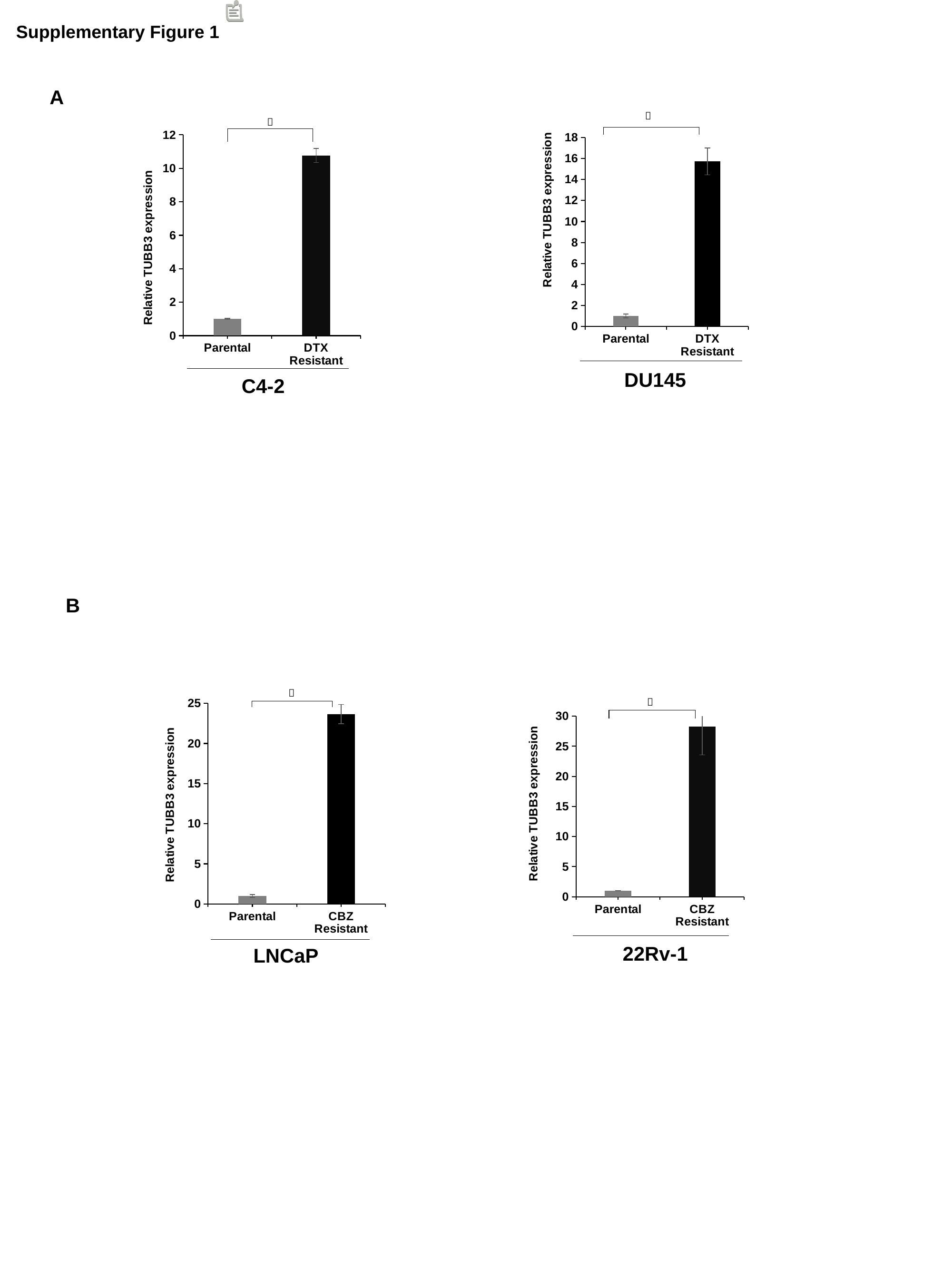

Supplementary Figure 1
A
＊
### Chart
| Category | |
|---|---|
| Parental | 1.0 |
| DTX
Resistant | 15.725214665385131 |DU145
＊
### Chart
| Category | |
|---|---|
| Parental | 1.0 |
| DTX
Resistant | 10.775373921748765 |C4-2
B
＊
### Chart
| Category | |
|---|---|
| Parental | 1.0 |
| CBZ
Resistant | 23.662037586677997 |LNCaP
＊
### Chart
| Category | |
|---|---|
| Parental | 1.0 |
| CBZ
Resistant | 28.303340534394458 |22Rv-1
