# Supplementary material for: TUBB3 Reverses Resistance to Docetaxel and Cabazitaxel in Prostate Cancer
Source: Int J Mol Sci. 2019 Aug 13;20(16):3936. doi: 10.3390/ijms20163936 (PMC6719236; doi:10.3390/ijms20163936)
Supplement: Supplementary file 1 [file ijms-20-03936-s001.zip › ijms-567404 sp for proof/Supplementary table 1 28 sample_CLEAN.docx]

| **Supplementary Table 1.** Clinicopathological characteristics  of the patients with prostate cancer. | | | |
| --- | --- | --- | --- |
| **Sample** | **PSA (ng/mL)** | **TNM stage** | **Gleason score** |
| 1 | 10.5 | T3aN0M0 | 3+4, ter.5 |
| 2 | 494 | T3bN0M1b | 5+4 |
| 3 | 7.3 | T3aN0M0 | 3+4 |
| 4 | 71.7 | T3bN0M1b | 4+4 |
| 5 | 4.4 | T2bN0M0 | 3+4 |
| 6 | 8.6 | T2aN0M0 | 4+3 |
| 7 | 7.2 | T2bN0M0 | 3+4 |
| 8 | 12 | T2bN0M0 | 4+4 |
| 9 | 8.1 | T2bN0M0 | 4+4 |
| 10 | 8.4 | T2bN0M0 | 3+3 |
| 11 | 7.7 | T3aN0M0 | 3+4 |
| 12 | 4.8 | T2bN0M0 | 4+5 |
| 13 | 4.4 | T2bN0M0 | 3+3 |
| 14 | 11.2 | T2cN0M0 | 5+4 |
| 15 | 22.5 | T2bN0M0 | 4+3 ter.5 |
| 16 | 6.4 | T2bN0M0 | 4+4 |
| 17 | 4.6 | T2cN0M0 | 4+3 |
| 18 | 18.1 | T3bN0M0 | 4+5 |
| 19 | 5.6 | T2cN0M0 | 4+3 |
| 20 | 10.4 | T2cN0M0 | 4+5 |
| 21 | 43.1 | T3bN0M0 | 4+5 |
| 22 | 28.5 | T2cN0M0 | 5+3 |
| 23 | 9.2 | T3aN0M0 | 4+5 |
| 24 | 11.6 | T2cN0M0 | 4+3 |
| 25 | 8.9 | T2bN0M0 | 3+4 |
| 26 | 5.9 | T2bN0M0 | 3+4 |
| 27 | 15.1 | T2cN0M0 | 4+3 |
| 28 | 12.6 | T2cN0M0 | 4+5 |

PSA: prostate-specific antigen.
